# Delays in Time to Head and Neck Cancer Treatment: A South Australian Perspective

**DOI:** 10.3390/medicina58020145

**Published:** 2022-01-18

**Authors:** Lachlan Cook, Charmaine Woods, Tracey Nicholls, Eng H. Ooi

**Affiliations:** 1 Department of Otorhinolaryngology, Head and Neck Surgery, Flinders Medical Centre, Adelaide 5042, Australia; charmaine.woods@flinders.edu.au (C.W.); tracey.nicholls@sa.gov.au (T.N.); eng.ooi@flinders.edu.au (E.H.O.); 2 Flinders Health and Medical Research Institute, College of Medicine and Public Health, Flinders University, Adelaide 5042, Australia

**Keywords:** head and neck neoplasms, time to treatment

## Abstract

*Background and Objectives:* In head and neck cancer, delays in time to treatment are associated with poorer clinical outcomes. Within Australia, it is recommended that primary treatment is initiated within 56 days of initial referral. The aim of this study was to assess whether head and neck cancer treatment was delivered within these timeframe guidelines at our institution and identify factors associated with treatment delays. *Methods:* This retrospective cohort study assessed patients newly diagnosed with head and neck cancer over a 24 months period (2018 to 2019) at Flinders Medical Centre, Australia. Time to treatment intervals were calculated for comparison to local timeframe guidelines. *Results:* A total of 72 patients met the inclusion criteria. The median time from specialist referral to treatment initiation was 45.5 days (IQR 29–61), with 72% meeting the 56 days guideline. On univariate logistic regression, patients undergoing primary radiotherapy treatment were less likely to meet this guideline than those undergoing primary surgery (OR 8.8, 95% CI 2.6–28.9, *p* < 0.001), as were those requiring prophylactic gastrostomy tube insertion (OR 3.1, 95% CI 1.1–9.0, *p* < 0.05). Treatment initiation beyond 56 days had no significant impact on 12 months overall survival or disease-free survival. *Conclusions*: The findings of this study demonstrate that primary radiotherapy treatment is associated with delays in head and neck cancer treatment initiation, likely related to time consuming pre-treatment factors such as gastrostomy tube insertion.

## 1. Introduction

Head and neck cancer has the seventh highest incidence and mortality rate of malignancies worldwide [1]. The time taken to commence treatment for head and neck cancer is important, where delays can result in worse oncologic outcomes [2,3]. In a systematic review of the effects of increased interval to treatment commencement, Schutte et al. showed that treatment delays are associated with higher cancer staging and worse survival outcomes [2]. Similarly, Murphy et al. performed a retrospective cohort study assessing the impact of treatment delay on median overall survival and showed that prolonged time to treatment initiation was an independent predictor of worse mortality, particularly when time to treatment initiation exceeded 60 days [3].

In Australia, the recommended timeframes in which head and neck cancer treatment should be planned and initiated are set out in the guideline “Optimal care pathway for people with head and neck cancer” [4]. Its recommendation is that curative intent treatment be initiated within 56 days of initial referral. This comprises of a 14 days window from referral to specialist appointment, 14 days from specialist appointment to multidisciplinary team meeting, and 28 days from the treatment decision to treatment initiation, regardless of whether the primary treatment modality is radiotherapy or surgery. Where surgery is the primary treatment modality, adjuvant radiotherapy should be commenced within 42 days of surgery.

Flinders Medical Centre (FMC) is a public tertiary referral centre in Adelaide, Australia. The FMC Head and Neck Multidisciplinary Team (MDT) is a weekly consultant led meeting with representatives from surgery, radiation oncology, medical oncology, radiology, pathology, allied health with dietetics, speech pathology and nursing. The MDT makes consensus recommendations on treatment options which are then presented to patients for decision.

This study aimed to assess whether head and neck cancer treatment was initiated in a timely manner at FMC, and to identify factors associated with a delay in treatment initiation.

## 2. Materials and Methods

A single-centre retrospective cohort study was performed of all patients discussed at the FMC Head and Neck MDT between 1 January 2018 and 31 December 2019. Inclusion criteria consisted of patients with a new primary head and neck cancer diagnosis who were referred to the Department of Otorhinolaryngology, Head and Neck Surgery at FMC, that underwent curative intent treatment. Exclusion criteria included patients presenting with recurrent or residual disease, non-head and neck primary malignancy, benign disease, referral to and work up by another surgical department, workup and/or treatment in the private health system, or the patient receiving treatment in another public hospital network.

For those cases which met the inclusion criteria, the following data were extracted from medical record case notes: age in years; gender; residential remoteness classified as metropolitan (Australian Bureau of Statistics Remoteness Area 1) or regional/remote (Australian Bureau of Statistics Remoteness Areas 2–5); anatomical tumour site; histopathological diagnosis and tumour stage (TNM AJCC 8th Edition); date of referral, clinic appointment, MDT meeting, treatment initiation, treatment completion, recurrence, death; treatment modality (primary surgery or primary radiotherapy); special needs dental review (required or not required); prophylactic gastrostomy tube (required or not required); and pre-treatment fluorodeoxyglucose positron emission tomography (PET) scan (required or not required). Dental review, gastrostomy tube and/or PET scan are not always required in the pre-treatment phase of head and neck cancer and were hypothesised to be factors affecting the time to treatment initiation. In the case where surgery is the primary treatment modality and adjuvant treatment is required, gastrostomy tube insertion and special needs dental may be provided in the timeframe between surgery and adjuvant treatment, hence not causing delay to treatment initiation.

Treatment time intervals were calculated using the following timepoint definitions: time of referral defined as the date a referral was received by the ENT outpatient clinic; date of specialist appointment was the first Head and Neck Clinic encounter; MDT date was the date the patient was first discussed at the Head and Neck Cancer MDT; treatment decision date was the MDT meeting date where a definitive treatment plan was made; treatment initiation date was dependent on primary treatment modality, either the first day of radiotherapy, or the date of surgery; the date of adjuvant treatment commencement was recorded as the first day of radiotherapy subsequent to completion of surgery. Treatment completion date was either the day of surgery in unimodality surgical treatment or the final day of radiotherapy where primary or adjuvant radiotherapy was used. Treatment time intervals were calculated for each case, compared against the recommended treatment timeframes, and recorded as either meeting or not meeting the “Optimal care pathway for people with head and neck cancer” guideline. One-year overall survival and disease-free survival were calculated, respectively, as the proportion of patients alive and the proportion of patients alive without locoregional or distant recurrence at 12 months after commencement of treatment.

Categorical variables are presented as count (%) and proportions analysed using chi-square. Time interval and survival data were not normally distributed and presented as median and interquartile range (IQR) or range, with analyses utilising non-parametric Mann–Whitney tests. Logistic regression was utilised to identify factors predictive of not meeting the time to treatment guidelines. Factors with *p* < 0.2 in a univariate logistic regression model were included for multivariate analysis. Statistical significance considered with *p* < 0.05. Statistical analyses were conducted using IBM SPSS Statistics for Windows, version 27.0. Figures were generated using GraphPad Prism 9.

## 3. Results

**Figure 1:**
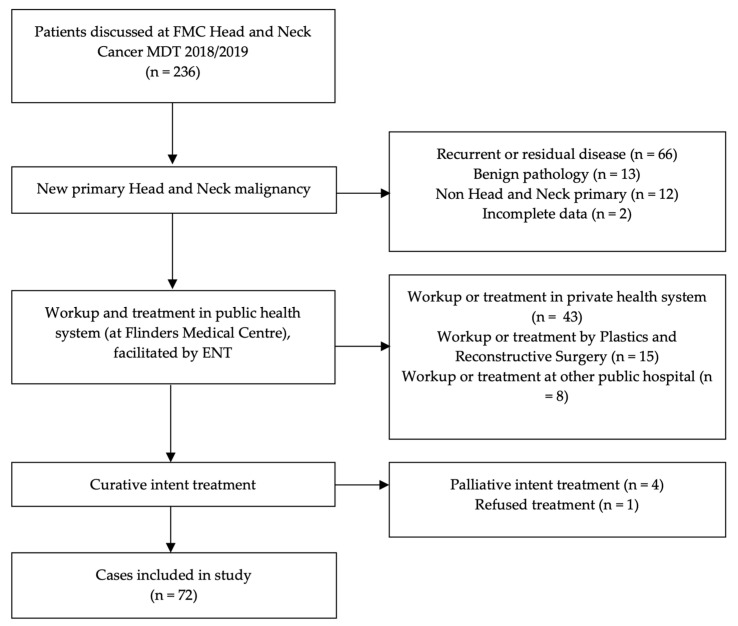
Study data set detailing number and reason for patients excluded from analysis.

A total of 236 patients were discussed at the Head and Neck Cancer MDT between January 2018 and December 2019. A total of 72 patients met the criteria for inclusion in this study (Figure 1).

The median age of patients was 65 years (IQR 54–75). A 71% (51/72) male predominance was seen, with 75% (54/72) of patients residing in metropolitan Adelaide. Tumour characteristics and treatment modality are presented in Table 1. Malignancy of the oropharynx was most common (40%, 29/72), followed by salivary gland, unknown primary, and oral cavity, with 82% (60/72) being squamous cell carcinomas (SCC). Stage I (25/72) and stage IV (23/72) malignancies were the most common. The treatment modality was primary surgery for 60% (43/72) of cases.

A pre-treatment special needs dental review was required in 74% (53/72) of all cases, a prophylactic gastrostomy tube was inserted in 40% (29/72) of patients, and a pre-treatment PET scan was required in 47% (34/72) of cases. A higher proportion of patients undergoing primary radiotherapy treatment required pre-treatment dental review (89% radiotherapy vs. 63% surgery; *p* < 0.05), prophylactic gastrostomy tube (64% radiotherapy vs. 23% surgery; *p* < 0.05) or PET scan (68% radiotherapy vs. 35% surgery *p* < 0.05).

### 3.1. Timeframes to Treatment Initiation

The timeframes for head and neck cancer treatment initiation, including the percentage of cases meeting the recommended guideline, is presented in Table 2. The overall median timeframe from referral to treatment completion was 109.5 days (IQR 80–129 days).

### 3.2. Identification of Factors Associated with Time to Treatment Initiation

**Figure 2:**
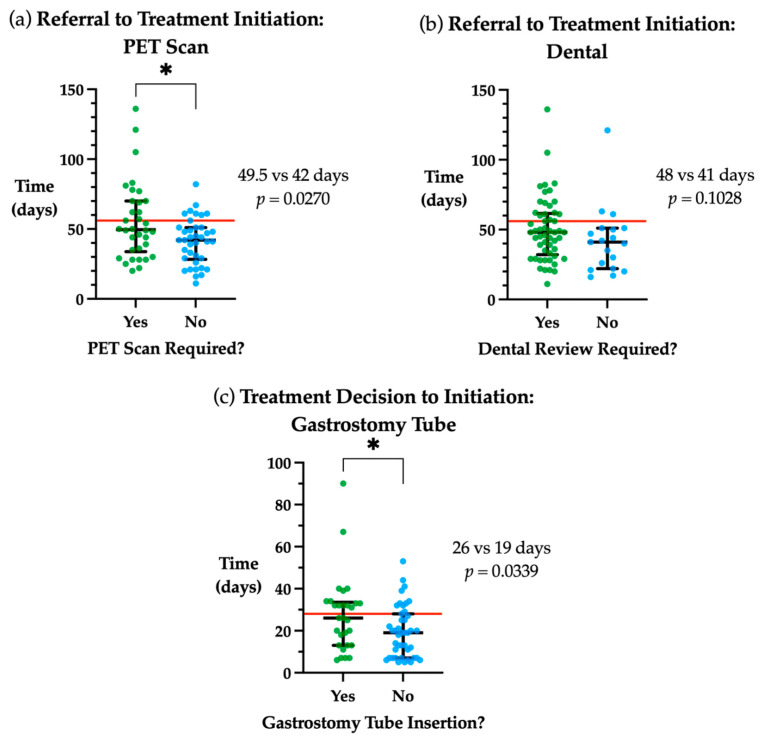
Factors associated with time to treatment initiation. (**a**) Days from referral to treatment initiation stratified by whether a PET scan was required. (**b**) Days from referral to treatment initiation stratified by whether a special needs dental review was required. (**c**) Days from treatment decision to initiation stratified by whether gastrostomy tube insertion was needed. Red line indicates guideline timeframe, 56 days from referral to treatment initiation and 28 days from treatment decision to initiation. Median and interquartile range plotted in black. * *p* < 0.05.

In cases where a PET scan was required during the pre-treatment workup phase, a 7.5 days delay was observed in the timeframe from referral to treatment initiation when requiring PET vs. those not needing a PET scan (*p* = 0.027, Figure 2a). The requirement for a dental review showed a trend towards a longer time from referral to treatment initiation (*p* > 0.05, Figure 2b). Those patients who required a gastrostomy tube had a longer treatment decision to initiation timeframe compared to those who did not (*p* = 0.034, Figure 2c). Patients with early-stage disease (AJCC 8th Edition Stage I/II) had treatment initiated a median of 10 days later than late stage (III/IV) (49 vs. 39 days, *p* = 0.011).

**Figure 3:**
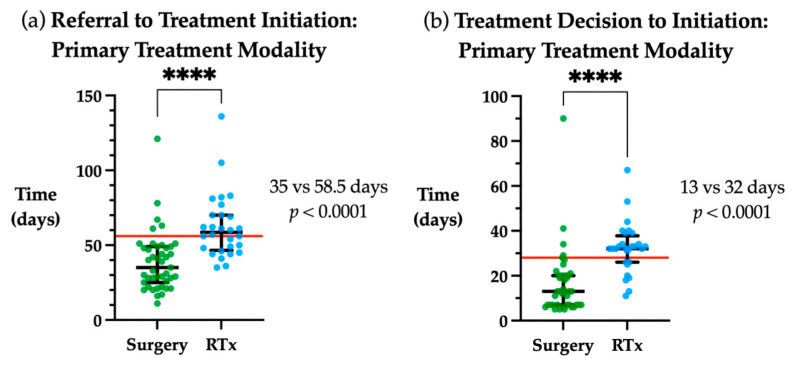
Time to treatment initiation by primary treatment modality. (**a**) Days from referral to treatment initiation for primary surgical or radiotherapy (RTx) treatment modality. (**b**) Days from treatment decision to initiation for primary surgical or radiotherapy treatment modality Red line indicates guideline timeframe, 56 days from referral to treatment initiation and 28 days from treatment decision to initiation. Median and interquartile range plotted in black. **** *p* < 0.0001.

There were significant differences in time to treatment initiation based on primary treatment modality. When assessing the overall timeframe between initial referral to commencement of treatment, initiation of primary radiotherapy was a median of 23.5 days longer than primary surgery (*p* < 0.0001, Figure 3a). Similarly, the time from treatment decision to initiation was a median of 19 days longer in primary radiotherapy vs. primary surgery (*p* < 0.0001, Figure 3b).

Assessing timeframes from referral to primary treatment initiation, the factors of whether a PET scan, gastrostomy tube insertion or special needs dental review was required reveals a trend that each these factors increase time to treatment in primary radiotherapy treatment, but not in primary surgical treatment (Table 3).

### 3.3. Identifying Factors Predictive of Exceeding the Referral to Treatment Initiation Timeframe

Logistic regression modelling was utilised to identify specific factors that could predict exceeding the guideline timeframe from referral to treatment initiation (Table 4).

On univariate logistic regression modelling for the timeframe between referral and treatment initiation, patients needing gastrostomy tube insertion were 3.1-fold more likely to not meet the 56 days guideline (*p* < 0.05), but the need for a PET scan or a special needs dental review were not predictive of treatment initiation beyond 56 days. Primary treatment modality was predictive of not meeting the 56 days guideline, with patients undergoing primary radiotherapy being 8.8-fold more likely not meet this guideline when compared with primary surgical treatment (*p* < 0.001). Combining the factors of stage, requiring a PET scan, gastrostomy tube and dental review did not produce a significant multivariate logistic regression model (*p* = 0.085) suitable to predict exceeding the recommended timeframe from referral to treatment initiation.

### 3.4. Mortality and Recurrence

At 12 months, disease-free survival was 84.7% (61/72) and overall survival was 90.3% (65/72). For those that met vs. those that exceeded the referral to treatment guideline there was no significant difference in disease-free survival (80.7% vs. 95%; *p* > 0.05) or overall survival (86.5% vs. 100%; *p* > 0.05).

## 4. Discussion

A delay in initiation of head and neck cancer treatment can have poor outcomes for patients. The aim of this study was to determine the timeframes in which head and neck cancer treatment is delivered via a MDT approach at Flinders Medical Centre. Time to treatment was assessed against the Australian treatment time guidelines and factors predictive of delays to treatment initiation were identified, including treatment modality and the need for gastrostomy insertion, at our institution.

A MDT approach to head and neck cancer management has been widely adopted, as it allows better adherence to best clinical practice, and reduces the time to treatment initiation [5,6]. The current study found that 67% of patients had treatment commenced within the 28 days recommended timeframe from treatment decision to initiation. For the overall time from referral to treatment commencement, the 56 days guideline was met for 72% of patients. The importance of meeting these recommended times to treatment initiation are highlighted in current literature, where delays to treatment in head and neck cancer were associated with worse oncologic outcomes [2,7]. Despite treatment delays in the current cohort not being significantly associated with worse 12-month mortality rates, larger studies have demonstrated clear associations between delays to treatment and decreased overall survival emerging as early as 18 months post treatment [3,8,9,10]. In a study of over 50,000 patients, Murphy et al. [3] showed prolonged time to treatment independently increased head and neck SCC mortality risk. The median overall survival when treatment was initiated in less than 52 days was 71.9 months, compared with 46.6 months where treatment was initiated in greater than 67 days.

The treatment timeframes outlined in the “Optimal care pathway for people with head and neck cancers” [4] are produced by the Cancer Council’s Head And Neck Cancers Working Group and are endorsed by the Australian Government. While not mandatory, they provide targets for clinicians and head and neck cancer treating teams. They are based on expert opinion without referencing primary research specifically on treatment times; however, the timeframes closely mirror the National Health Service’s (NHS) government-mandated cancer waiting times in the United Kingdom [11]. The guidelines in the UK are a maximum 14 day wait between referral and specialist appointment, 31 days between treatment decision and primary treatment initiation and 62 days between referral and treatment initiation. The respective guidelines in Australia are 14, 28 and 56 days. As such, the Australian guidelines are achievable and should be adhered to as closely as possible.

The median wait time of 45.5 days from referral to treatment initiation in this study is comparable to that presented in the local and international literature. In Australia, Flukes et al. [12] reported a median time of 61 days using the same timeframe definition, while Connell et al. [13] reported a mean time of 39.4 days from first ENT consultation to treatment initiation, which did not incorporate the time from referral to first consultation corresponding to the Australian guideline. International studies have largely reported the timeframe from histopathological diagnosis to treatment initiation, without reporting timing of referral or first specialist appointment. Using this alternative timeframe from diagnosis to treatment initiation (corresponding to 28 days in the Australian guideline), van Harten et al. [14] reported a median time of 37 days in the Netherlands, Murphy et al. [15] reported a median of 26 days in the United States and Polesel et al. [10] reported a median of 28 days in Italy.

Factors hypothesised to be associated with treatment delays at our institution were the requirement for a PET scan, a dental review and gastrostomy tube insertion, due to limited availability and associated logistical challenges. Needing a PET scan or gastrostomy tube insertion were significantly associated with longer times to treatment initiation, with those requiring gastrostomy tube insertion being 3-fold more likely to not meet the time to treatment guideline, compared to patients not requiring a PEG.

PET imaging plays an important role in the pre-treatment phase for head and neck cancer. It is useful to assess for a primary tumour in patients with cervical lymph node metastasis from carcinoma of unknown origin, as well as cervical lymph node assessment in newly diagnosed head and neck cancer [16]. The PET scan may also detect distant metastatic disease or second primary tumours. This allows for more accurate cancer staging, which has prognostic implications, thus PET findings may impact the extent, intensity, and modality of planned treatment. Given we found treatment was initiated a median of 7 days later when a PET scan was required, its requirement in pre-treatment workup should be identified as early as possible, such that it can be available at the time of MDT to allow for timely, informed treatment decision making.

Gastrostomy feeding tubes are frequently required in patients undergoing chemoradiotherapy for head and neck cancer, due to treatment related toxicities of mucositis, odynophagia and dysphagia [17]. Impaired swallowing function associated with these side effects can lead to significant weight loss, dehydration, malnutrition and aspiration events. Gastrostomy tubes can be inserted prophylactically for patients deemed at risk of these complications, or reactively when debilitating swallowing symptoms arise after treatment has commenced. The timing of gastrostomy tube placement remains somewhat controversial, given prophylactically inserted gastrostomy tubes may not need to be used throughout the course of treatment, and may be associated with long term gastrostomy tube dependence [17,18]. At this institution, prophylactic gastrostomy tubes are inserted for high-risk patients, including T4 or hypopharyngeal tumours planned for chemoradiotherapy; T3 tumours of the oral cavity, oropharynx or larynx; bilateral radiotherapy planned; major surgery planned; significant dysphagia or severe malnutrition pre-treatment. This is in keeping with evidence showing prophylactic insertion is associated with decreased malnutrition rates, significantly fewer hospitalisations and improved quality of life [18,19,20]. It should be noted, however, that prophylactic insertion has not shown any survival benefit [18].

In the current study, prophylactic gastrostomy tube insertion was associated with a prolonged time from treatment decision to initiation, and was an independent risk factor associated with not meeting the referral to treatment initiation guideline. When a pre-treatment gastrostomy tube is deemed necessary, it is important to advocate for prompt insertion, such that its benefits are not offset by delaying the commencement of treatment.

Special needs dental review is required prior to radiotherapy, to maximise quality of life following oncological treatment and plan prophylactic dental extractions to reduce the risk of osteoradionecrosis [21]. This study demonstrated treatment was initiated a median of 7 days later when a dental review was required. These findings highlight the value of having an accessible on-site dental service to facilitate urgent review of patients with head and neck cancer, such that radiotherapy can commence in a timely manner. It should be noted that in the case of primary surgical treatment with adjuvant radiotherapy, dental review can occur after surgery is completed, hence not impacting the time to treatment initiation.

When assessing the effect primary treatment modality had on time to treatment initiation, there were significant differences between primary surgery compared with primary radiotherapy. The median time difference was 19 days longer for primary radiotherapy between treatment decision and initiation, and 23.5 days from referral to commencement of treatment. Furthermore, primary radiotherapy has an 8 fold increased risk of not meeting the 56 day referral to treatment initiation guideline compared to patients undergoing primary surgery treatment. Given that patients undergoing primary radiotherapy were much more likely to need a PET scan, gastrostomy tube insertion and/or a pre-treatment dental review, the requirement of these factors are likely to be the key determinants in treatment delays. As these factors are essential for accurate diagnosis and the delivery of safe treatment, when they are required, we hypothesise that early, urgent referrals are likely to prevent delays translating into longer intervals to treatment initiation. When surgery is the primary treatment modality and adjuvant radiotherapy is planned, special needs dental review and prophylactic gastrostomy insertion can occur after surgery is completed, hence these factors are less likely to impact the time to primary treatment initiation.

The finding that primary treatment modality impacts time to treatment initiation is consistent with the published literature, where radiotherapy has been associated with longer times to commencement of treatment [7,13,22]. Systematic reviews of time to treatment in head and neck cancer reveals that research is predominantly focused on the impact of delays on patient outcomes, but studies have also identified race, socioeconomic status, treatment facility type, tumour site and patient comorbidities as potential factors delaying treatment initiation [2,7,12,22]. The pre-treatment factor of requiring gastrostomy insertion has not previously been identified with delay to treatment initiation [7,22]. A key strength of this study is that the studied timeframes are compared against published treatment time guidelines [4], to assess whether these recommended timeframes are being met. To our knowledge, there are no other studies which compare time to treatment initiation against guidelines, including the recently published Australian studies assessing time to initiation of head and neck cancer treatment [12,13]. Further to this, incorporating the time from general practitioner referral to specialist head and neck clinic provides a more holistic view of the patient journey, which is not consistently captured in the current literature.

Limitations of this study include the small sample size of 72 patients, and a limited follow up duration of 12 months for mortality and recurrence data, with longer follow up expected to demonstrate a significant impact on overall survival and disease-free survival. Future prospective studies evaluating a larger sample size using our methodology coupled with a 5 year follow up duration would allow for a more precise account of how the demonstrated delays to treatment initiation affect the clinically relevant outcomes of mortality and recurrence rates, as well as controlling for variables such as cancer stage and site. This is important for addressing the factors in the healthcare system from primary care referral to treatment initiation and completion of treatment. Other future directions include implementation of a streamlined urgent referral process for dental review, PET scan and gastrostomy tube insertion prior to treatment initiation. Measurement of time to treatment initiation pre and post implementation of such a process would be useful to assess any reduction in timeframes, correlating with improved clinical outcomes.

## 5. Conclusions

This study has demonstrated that primary radiotherapy treatment is associated with delays in head and neck cancer treatment initiation, likely related to time consuming factors during the pre-treatment phase such as obtaining a PET scan, requiring review by the special needs dental service and prophylactic insertion of a gastrostomy tube.

## Figures and Tables

**Table 1:** Tumour characteristics and treatment modality.

Tumour Site	*n*	%
Oropharynx	29	40.0
Salivary gland	12	16.7
Unknown primary	9	12.5
Oral	8	11.1
Larynx	6	8.3
Skin	3	4.2
Hypopharynx	2	2.8
Nasopharynx	1	1.4
Thyroid	1	1.4
Sinonasal	1	1.4
**Histopathology**		
Squamous cell carcinoma	60	83.3
Basal cell carcinoma	2	2.8
Mucoepidermoid carcinoma	2	2.8
Nasopharyngeal carcinoma	1	1.4
Adenoid cystic carcinoma	1	1.4
Basal cell adenocarcinoma	1	1.4
Salivary duct carcinoma	1	1.4
Secretory carcinoma	1	1.4
Alveolar soft part sarcoma	1	1.4
Acinic cell carcinoma	1	1.4
Merkel cell carcinoma	1	1.4
**Stage (AJCC 8th Edition)**		
I	25	34.7
II	9	12.5
III	15	20.9
IV	23	31.9
**Treatment Modality**		
Primary surgery	43	59.7
Surgery alone	15	20.8
Surgery + radiotherapy	18	25.0
Surgery + chemoradiotherapy	9	12.5
Surgery + chemotherapy	1	1.4
Primary radiotherapy	29	40.3
Radiotherapy alone	4	5.6
Chemoradiotherapy	25	34.7

**Table 2:** Timeframes for head and neck cancer treatment at Flinders Medical Centre in 2018 and 2019 compared to Australian guidelines.

Timeframe	Days Median (IQR)	Guideline Recommendation (Days)	Met Guideline *n* (%)
Referral to Head and Neck Clinic	12.5 (3–20)	14	40/72 (56%)
Head and Neck Clinic to MDT	7 (3–14)	14	56/72 (78%)
Treatment Decision to Initiation	20 (11–32)	28	48/72 (67%)
Treatment Decision to Primary Surgery	13 (7–20)	28	39/43 (91%)
Treatment Decision to Primary Radiotherapy	32 (26–38)	28	8/28 (29%)
Referral to Treatment Initiation	45.5 (29–61)	56	52/72 (72%)
Referral to Primary Surgery	35 (25–49)	56	38/43 (88%)
Referral to Primary Radiotherapy	58.5 (47–70)	56	13/28 (46%)
Primary Surgery to Adjuvant Radiotherapy	42 (37–48)	42	16/29 (55%)

**Table 3:** Time from referral to treatment initiation for primary treatment modalities.

	Referral to Treatment Initiation (Median Days)								
	PET Scan			Gastrostomy Tube			Special Needs Dental		
	No ^1^	Yes	*p* Value	No	Yes	*p* Value	No	Yes	*p* Value
**Primary Surgery**	37.5	30	0.78	40	28.5	0.32	41	33	0.69
**Primary Radiotherapy**	56	62	0.28	55	61	0.52	41	61	0.002

^1^ ‘No’ and ‘Yes’ refers to whether each factor (PET, gastrostomy tube or dental) was required.

**Table 4:** Univariate logistic regression findings for evaluating factors predictive of not meeting the 56 day guideline from time of referral to treatment initiation.

	Odds Ratio (95% CI)	*p* Value
**Gender**	1.462 (0.484–4.410)	0.501
**Histopathology ^1^**	2.143 (0.426–10.779)	0.355
**Residential Remoteness**	2.297 (0.586–9.006)	0.233
**Stage ^2^**	2.045 (0.716–5.846)	**0.182** ^3^
**PET**	2.741 (0.938–8.016)	**0.065**
**Gastrostomy Tube**	3.088 (1.064–8.966)	**0.038**
**Special Needs Dental**	2.519 (0.646–9.826)	**0.184**
**Primary Treatment Modality**	8.769 (2.662–28.884)	**<0.001**

^1^ Histopathology classified as SCC vs. other. ^2^ Stage classified as late (AJCC 8th Stage III/IV) vs. early (I/II). ^3^ Bold *p* values indicate factors included in multivariate analysis.

## Data Availability

Data is available on request from the corresponding author.
